# New developments in gold-catalyzed manipulation of inactivated alkenes

**DOI:** 10.3762/bjoc.9.294

**Published:** 2013-11-21

**Authors:** Michel Chiarucci, Marco Bandini

**Affiliations:** 1 Department of Chemistry “G. Ciamician”, Alma Mater Studiorum – University of Bologna, via Selmi 2, 40126 Bologna, Italy

**Keywords:** alkene, gold catalysis, mechanism, organic synthesis

## Abstract

Over the recent years, the nucleophilic manipulation of inactivated carbon–carbon double bonds has gained remarkable credit in the chemical community. As a matter of fact, despite lower reactivity with respect to alkynyl and allenyl counterparts, chemical functionalization of isolated alkenes, via carbon- as well as hetero atom-based nucleophiles, would provide direct access to theoretically unlimited added value of molecular motifs. In this context, homogenous [Au(I)] and [Au(III)] catalysis continues to inspire developments within organic synthesis, providing reliable responses to this interrogative, by combining crucial aspects such as chemical selectivity/efficiency with mild reaction parameters. This review intends to summarize the recent progresses in the field, with particular emphasis on mechanistic details.

## Review

### 1 Introduction

Homogeneous gold catalysis is emerged as one of the most powerful means for the activation of C–C multiple bonds toward a number of complexity-oriented transformations. In this segment, gold-catalyzed addition of carbon- and heteroatom-based nucleophiles to inactivated alkenes are widely recognized as “capricious” transformations due to alkyne and allene counterparts [1–5]. However, over the past few years, tremendous developments were made, and some of the major contributes will be summarized in the present review.

**Figure 1:**
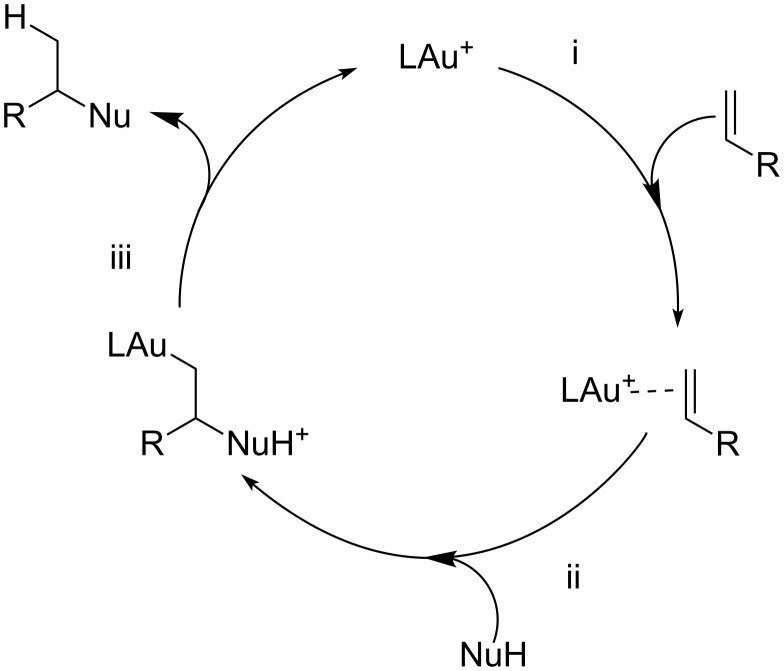
Elementary steps in the gold-catalyzed nucleophilic addition to olefins.

Mechanistically, it is generally accepted that the gold-catalyzed nucleophilic addition to alkenes proceeds through three elementary steps: i) activation of the C–C double bond by gold coordination. ii) *anti-*Nucleophilic attack with formation of an alkylgold intermediate (outer-sphere pathway). iii) Protodeauration with formation of the product and catalyst regeneration (Figure 1).

**Figure 2:**
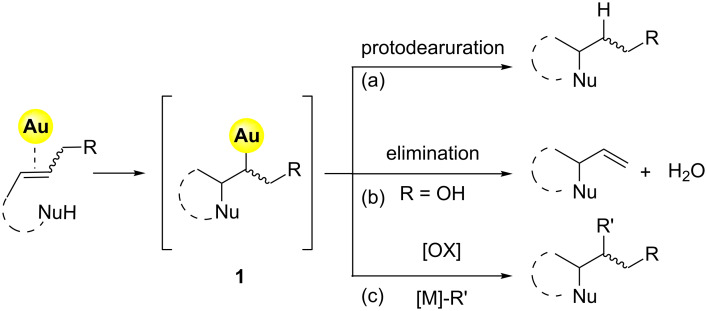
Different approaches for the gold-catalyzed manipulation of inactivated alkenes.

The simplified mechanistic sketch depicted in Figure 1 accounts also for the lower reactivity of alkenes with respect to different π-systems. In particular, the intrinsic inertness of alkylgold intermediate **1** (i.e. Csp^3^–Au bond) towards protodeauration (path a, Figure 2) determines the scarce reactivity of alkenes in nucleophilic addition reactions [6].

The use of allylic alcohols as C=C surrogates opens up an eliminative pathway for the cleavage of the C–Au bond in the intermediate **1** (Figure 2, path b). This reaction channel is accessible under mild conditions and without external activation when proper gold catalysts are employed [7–8].

Furthermore, in recent years, advances in the gold-catalyzed functionalization of alkenes rely on the use of “oxidative strategies” exploiting the [Au(I)/Au(III)] or [Au(I)Au(I)/Au(II)Au(II)] catalytic couples that can be accessed through the use of a suitable exogenous oxidant (Figure 2, path c) [9].

Last but not least, the potential role of Brønsted acid co-catalysis should always be considered when metal triflates are employed [10–11]. Indeed, it was demonstrated that catalytic amounts of TfOH could catalyze some specific additions of oxygen- and nitrogen-based nucleophiles to simple alkenes with comparable efficiency/selectivity as much as some metal triflates [12–13].

In this review some selected examples of gold-catalyzed nucleophilic additions to inactivated alkenes will be discussed with particular emphasis on the corresponding reaction machinery. In the context of this review, allylic alcohols will be treated as inactivated olefins, considering that normally activated equivalents, like halides, acetates, carbonates and phosphates are employed in metal-catalyzed Tsuji–Trost type alkylation [14–15].

### 2 Formation of C–O bonds

#### 2.1 Mechanistic considerations

The addition of oxygen-based nucleophiles to C–C multiple bonds is an effective and atom-economical process for the formation of new C–O bonds. In this direction numerous examples of metal and Brønsted-acid catalyzed condensations of alcohols, phenols and carboxylic acids to inactivated olefins have been reported [16].

Interestingly, He and co-workers compared the catalytic attitude of TfOH and PPh_3_AuOTf in the addition of various nucleophiles to alkenes, demonstrating how the gold complex and triflic acid can exhibit complementary efficiency [17]. This finding suggests that although Brønsted acid co-catalysis is a possible competing process, under suitable conditions the gold-catalyzed pathway is still the dominating process. For example by adopting the addition of *p-*NO_2_- and *p-*MeO-phenol to aryl and alkyl olefins as a model process, it emerged that TfOH was an effective catalyst at room temperature and led to decomposition of the starting material at higher temperatures. On the contrary, cationic [Au(I)] species exhibited comparable activity to TfOH at 85 °C (Table 1).

**Table 1:** Comparison of the catalytic activity of TfOH and PPh_3_AuOTf in the addition of phenols to alkenes.

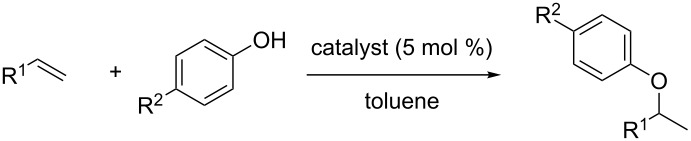					

Entry	R^1^	R^2^	*T* (°C)	Catalyst	

				TfOH	Ph_3_PAuOTf

1	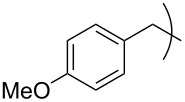	NO_2_	rt	93	–
2			85	trace	81
3	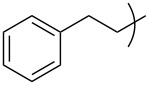	OMe	rt	57	–
4			85	trace	58

This complementary behavior can be ascribed to different activation modes of the double bond. Computational investigations on the [H^+^]-catalyzed reaction predicted a transition state with significant carbocation character [18–19], therefore more easily subjected to substrate isomerization or degradation. Notwithstanding, gold activation was shown to occur through the formation of a η^2^-complex [20–24].

**Figure 3:**
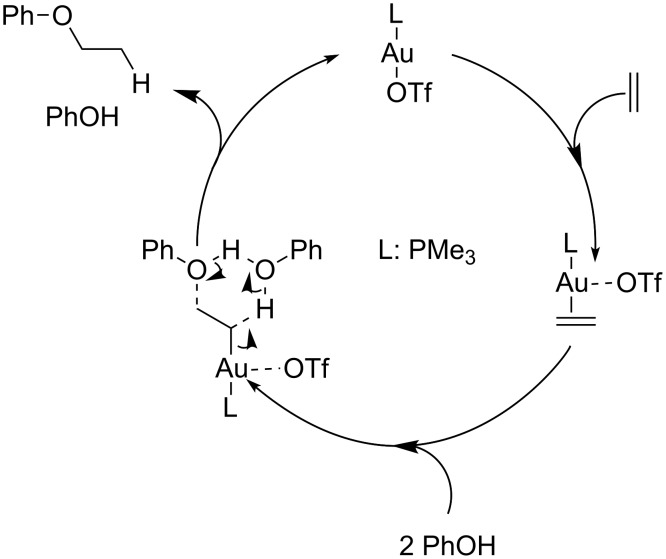
Computed mechanistic cycle for the gold-catalyzed alkoxylation of ethylene with PhOH.

An important contribution to this scenario, was also provided by Ujaque and co-workers, who performed a theoretical investigation on the Me_3_PAuOTf catalyzed addition of phenol to ethylene [25]. The proposed catalytic cycle initiated with the exchange of the TfO^−^ ion with the alkene reveals that the poorly coordinating anion seems to form a “loose” ion pair lying far from the metal center (Figure 3). This step was energetically favorable and the activation of the alkene by the gold cation was confirmed by elongation of the C–C double bond [26]. After an exhaustive survey of plausible reaction channels, it turned out that the phenol is involved in assisting the concerted addition/protodeauration machinery. The presence of a second molecule of phenol, acting as a proton shuttle, considerably lowered the energetic barrier for the protodeauration (i.e*.* rate limiting step of the overall process), which takes place through a more favorable 6-membered cyclic transition state. Analogous calculations indicate that also water is a potential proton transfer agent in the protodeauration event of the catalytic cycle.

#### 2.2 Selected examples

Initial reports dealing with the gold-catalyzed addition of oxygen-based nucleophiles to isolated olefins required the use of relatively acidic nucleophiles.

**Scheme 1:**
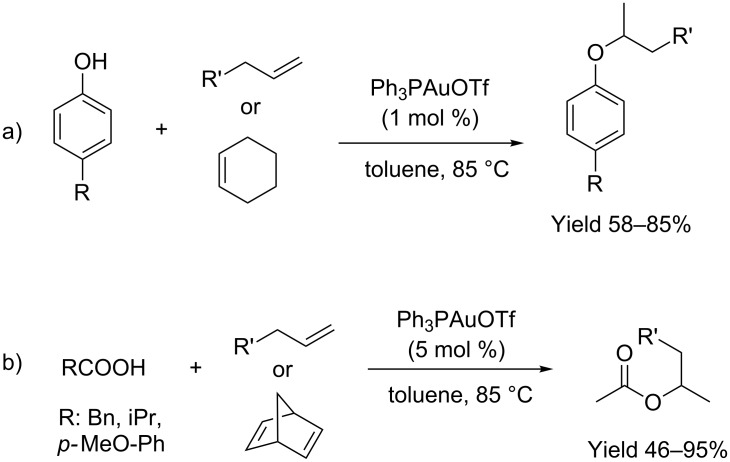
[Au(I)]-catalyzed addition of phenols and carboxylic acids to alkenes.

In their seminal works, Yang and He reported on the Ph_3_PAuOTf assisted Markovnikov-like addition of phenols and carboxylic acids to C=C under mild reaction conditions (Scheme 1) [27]. Electron-rich and electron-poor phenols reacted smoothly in toluene (85 °C) with only 1 mol % of catalyst loading (Scheme 1a). Contrarily, carboxylic acids required a higher loading of catalyst (5 mol %), but an acceptable yield was obtained even when sterically demanding 2-methylpropionic acid was employed as the nucleophile (Scheme 1b).

**Scheme 2:**
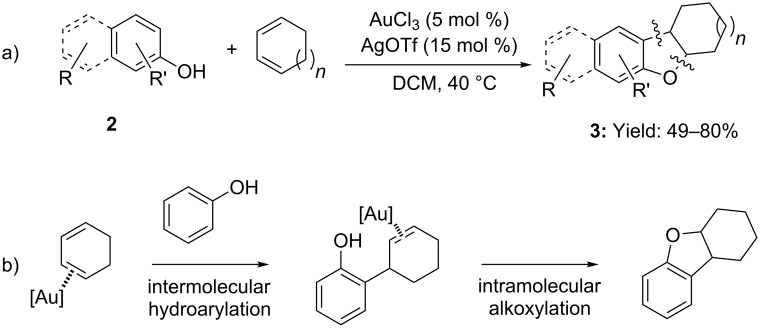
[Au(III)] catalyzed annulations of phenols and naphthols with dienes.

Li and co-workers exploited the [Au(III)]-catalyzed addition of phenols and naphthols to conjugated dienes realizing an efficient synthesis of dihydrobenzofuran derivatives **3** (Scheme 2a) [28]. The protocol was assumed to proceed via a two-step mechanism: involving an initial hydroarylation of the double bond followed by an intramolecular phenol addition (Scheme 2b).

**Scheme 3:**
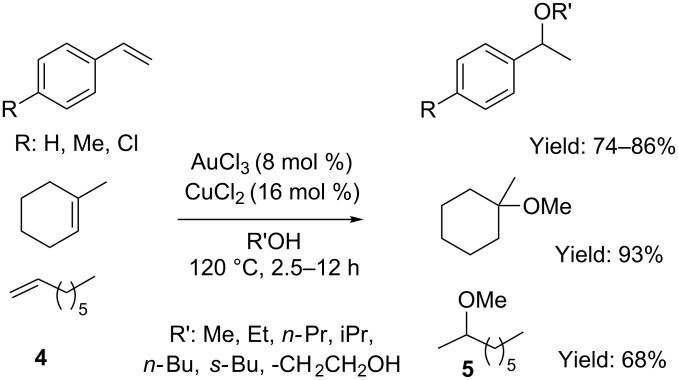
[Au(III)]-catalyzed addition of aliphatic alcohols to alkenes.

Shortly after, Zhang and Corma considerably expanded the scope of this transformation to aliphatic alcohols (Scheme 3) [29]. Under these conditions, the addition of primary and secondary alcohols to aryl and alkyl olefins **4** took place efficiently, with good yield and regioselectivity. However, poor diastereoselectivity was recorded in the presence of secondary alcohols.

The efficiency of the process relied on the stabilization of cationic [Au(III)] species by using a catalytic amount CuCl_2_ (16 mol %), which prevented gold deactivation via parasitic reductive side reactions [30–31].

**Scheme 4:**
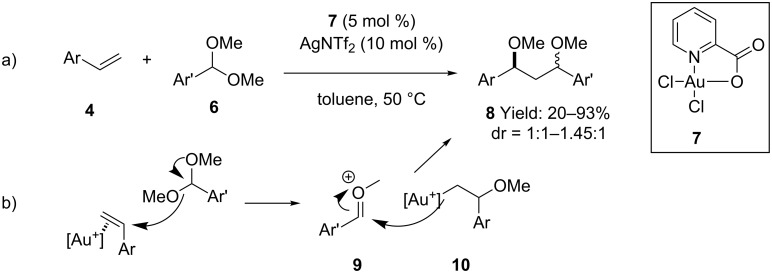
[Au(III)]-catalyzed carboalkoxylation of alkenes with dimethyl acetals **6**.

Moreover, recent advances in the alkoxylation of olefins enabled the use of simple dimethyl acetals **6** in the carboalkoxylation of alkenes (Scheme 4) [32]. A 1:2 mixture of [picAuCl_2_] (**7**) and AgNTf_2_ efficiently catalyzed the double functionalization of aryl alkenes **4** in good yields and mild conditions. Dialkyl substituted olefins afforded the product only in moderate yields whereas monoalkyl olefins were completely unreactive under the optimized conditions. Although an activation of the acetals by the gold catalyst cannot be ruled out, a reaction pathway involving gold activation of the alkene, followed by addition of the alkyl gold intermediate **10** to the activated carbonyl compound **9** was also hypothesized (Scheme 4b).

### 3 Hydroamination of olefins

#### 3.1 Mechanistic considerations

**Figure 4:**
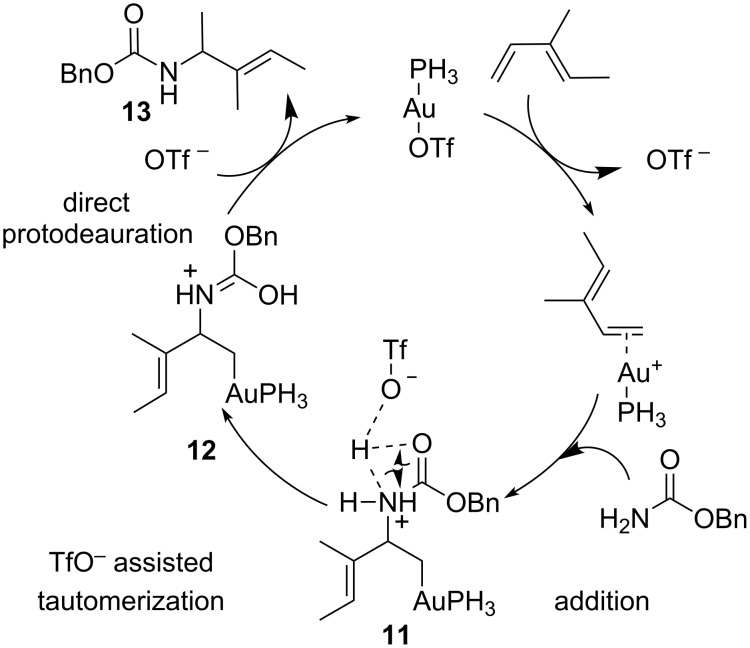
Postulated mechanism for the [Au(I)]-catalyzed hydroamination of olefins.

Due to the ubiquity of the C–N bond in organic compounds the development of efficient catalytic systems for the hydroamination of olefins is of particular significance from a practical point of view [33]. Although many metal and Brønsted acid assisted processes have been documented the high functional group tolerance of gold complexes combined with their high efficiency in the electrophilic activation of C–C multiple bonds have made gold catalysis an important tool for the hydroamination of alkenes. Detailed computational studies on the addition of benzyl carbamate to dienes revealed the protodeauration as the rate-determining step of the reaction [34]. Mechanistically, the initial stage was determined dealing with the energetically favourable substitution of the TfO^−^ ligand from the gold coordination sphere with the diene (Figure 4). Preferred coordination geometry is the η^2^-type, with the gold cation coordinating to a single double bond. Either direct coordination of the nucleophile to the gold cation, or reaction of the PH_3_AuOTf with the carbamate to deliver TfOH and PH_3_AuNHCOOBn [35] were ruled out by experimental and computational observations. Differently, addition of the nucleophile to the gold activated double bond took place with Markovnikov regioselectivity affording the intermediate **11**. At this point many possible reaction pathways were evaluated to shed light on the real mechanism of the protodeauration step. Interestingly the reaction profile with the lowest activation energy was distinguished in the TfO^−^ promoted tautomerization of **11** to form **12** followed by direct proton transfer to afford the product **13** and regeneration of the catalyst.

**Scheme 5:**
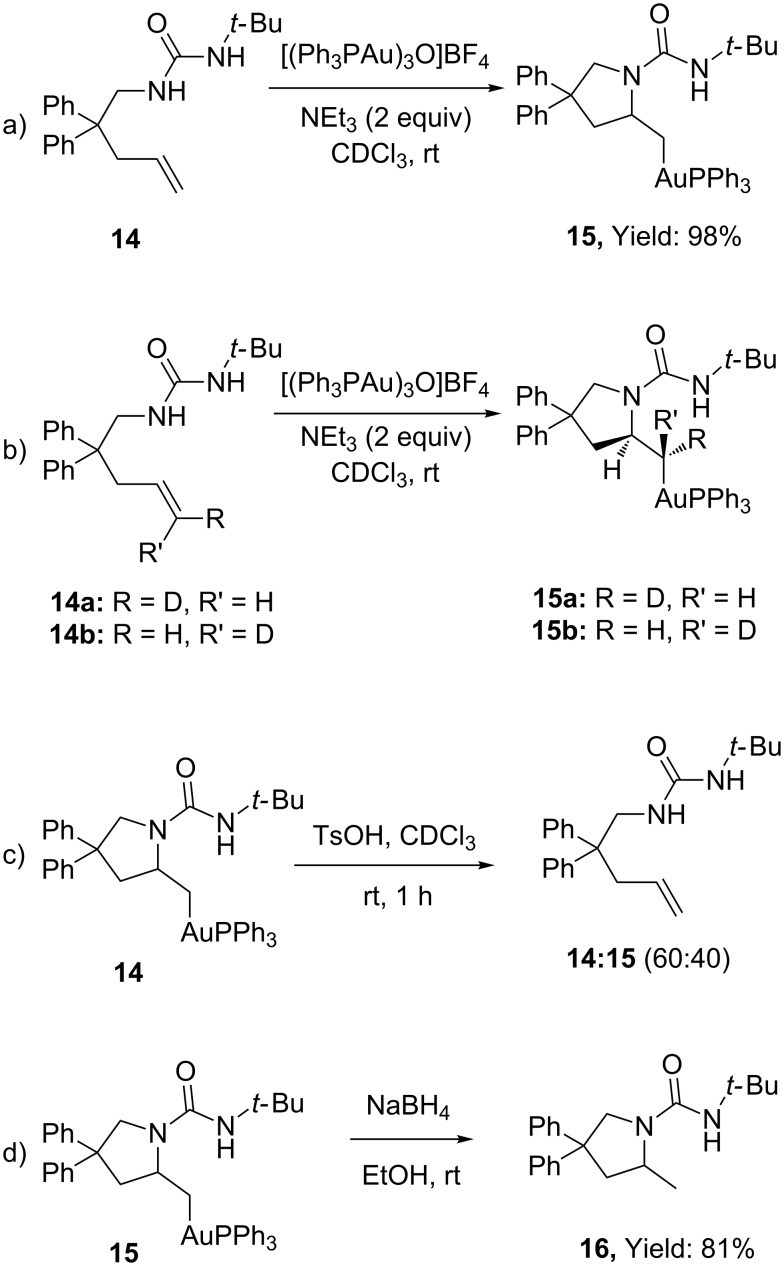
Isolation and reactivity of alkyl gold intermediates in the intramolecular hydroamination of alkenes.

Intermediates of type **11** were isolated by Toste in the intramolecular hydroamination of alkenyl urea **14** with a stoichiometric amount of phosphine–gold complex [(PPh_3_Au)_3_O]BF_4_ (Scheme 5a) [2]. The reaction proceeded at room temperature and was found being favoured by electron-withdrawing ligands. Finally, the use of the deuterated compounds **14a,b** permitted to confirm the *anti*-diastereoselective hydroamination of the double bond. (Scheme 5b). Interestingly, although **14** represents a likely intermediate of the reaction course, when it was treated with various Brønsted and Lewis acids, the expected product **16** was not observed and the elimination reaction to reform the starting material was the dominant process (Scheme 5c). Only reductive conditions afforded **16** in good yield (81%, Scheme 5d). This experimental evidence contributed substantially at the definition of the complex reaction machinery, with direct implication also in the correlated gold-catalyzed oxidative heteroarylation of unsaturated olefins (see section 6).

#### 3.2 Selected examples

**Scheme 6:**
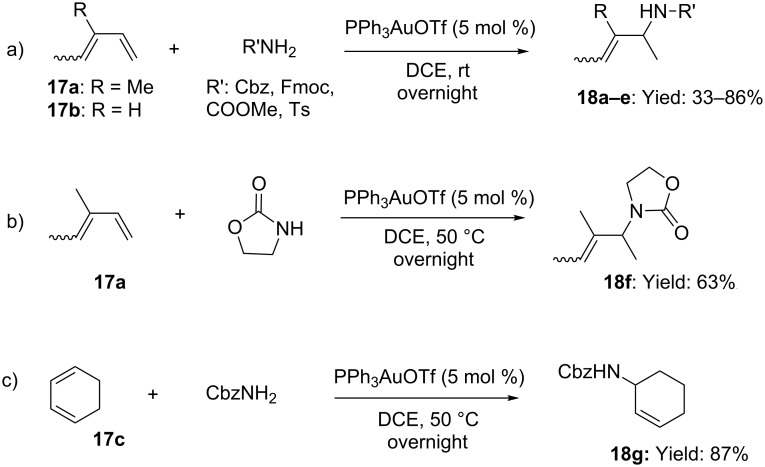
[Au(I)]-catalyzed intermolecular hydroamination of dienes.

Initial reports on the gold-catalyzed hydroamination of olefins were presented by He [36] and Widenhoefer [37], independently. He and Brouwer disclosed the intermolecular addition of carbamates, sulfonamides and imidazolidinones to linear and cyclic dienes **17** in the presence of catalytic amounts of PPh_3_AuOTf (Scheme 6). The method featured excellent 1,2-regioselectivity and high chemoselectivity, providing protected allylamines **18**, in good yields, using nearly equimolar amounts of the diene and nitrogen-based nucleophile.

**Scheme 7:**
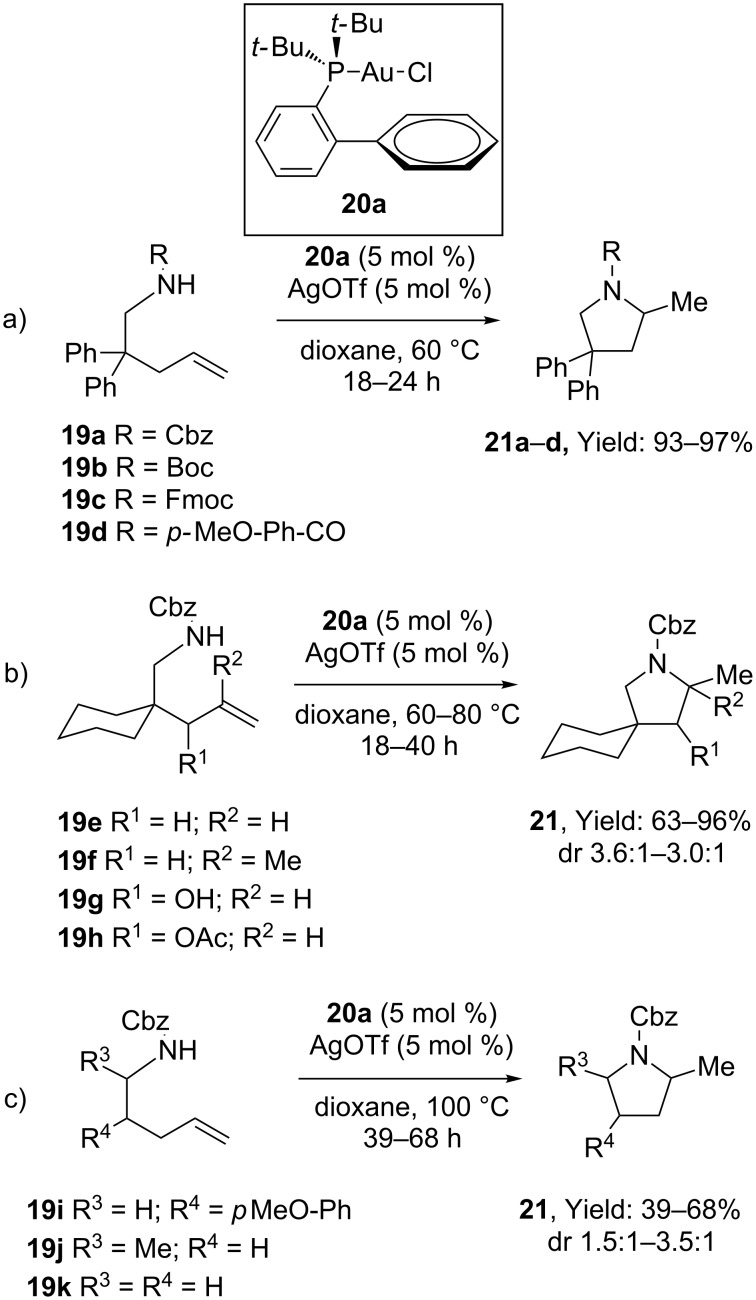
Intramolecular [Au(I)]-catalyzed hydroamination of alkenes with carbamates.

Analogously, Widenhoefer and Han developed the intramolecular addition of carbamates to terminal alkenes affording *N*-protected pyrrolidines and piperidines **21** (Scheme 7). Best conditions involved the use of a 1:1 mixture of [Au(I)] complex **20a** and AgOTf [38]. Although prolonged reaction times were required (up to 68 h in refluxing dioxane), remarkable tolerance in terms of carbamate protecting groups (Scheme 7a) and substituents at the carbon chain was recorded (Scheme 7b,c). Soon after the same team disclosed that the titled transformation could be extended to amide-based nucleophiles exploiting similar operational conditions [39].

**Scheme 8:**
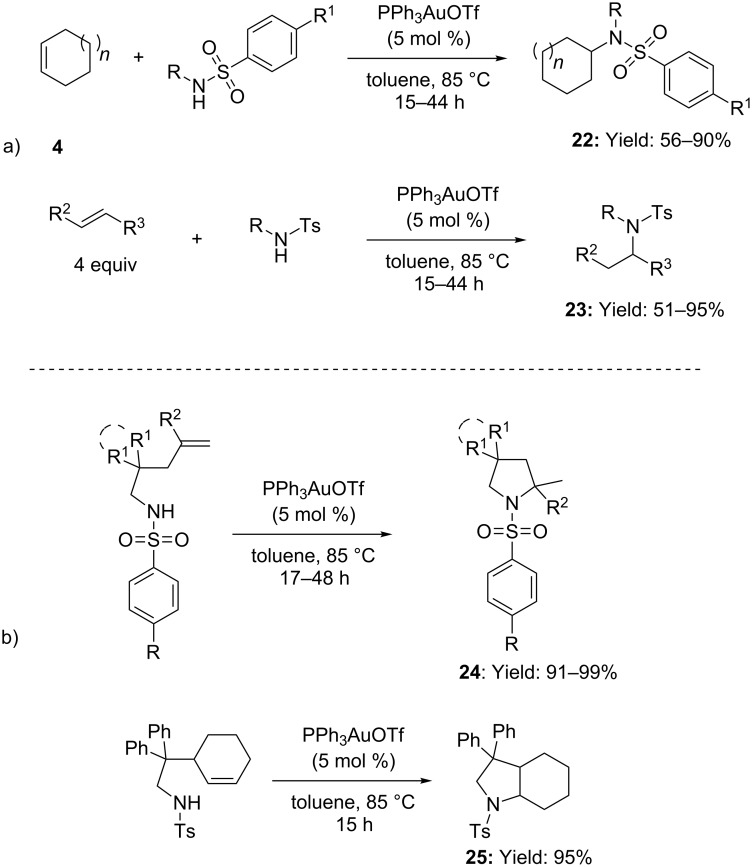
[Au(I)]-catalyzed inter- as well as intramolecular addition of sulfonamides to isolated alkenes.

After these seminal works the scope of gold-catalyzed hydroamination of olefins was extend to other classes of nitrogen nucleophiles. Sulfonamides were successfully employed in the intra- and intermolecular hydroamination of alkenes catalyzed by Ph_3_PAuOTf (toluene, 85 °C, Scheme 8) [40]. In particular, primary and secondary sulfonamides reacted smoothly with mono and disubstituted alkenes delivering nitrogen compounds **22** and **23** with Markovnikov regioselectivity (Scheme 8a). Moreover, *N-*protected pyrrolidines **24** and **25** were accessed by intramolecular addition in excellent yields (Scheme 8b).

The combined use of more electrophilic phosphite-gold complexes [41–42] and enabling techniques such as microwave irradiation [43] led to consistent improvement in efficiency of the catalytic system. In particular when (Ph_3_O)PAuOTf was employed under microwave irradiation with a catalyst loading as low as 0.05 mol %, the condensation of TsNH_2_ to norbornene was realized in quantitative yield.

**Scheme 9:**
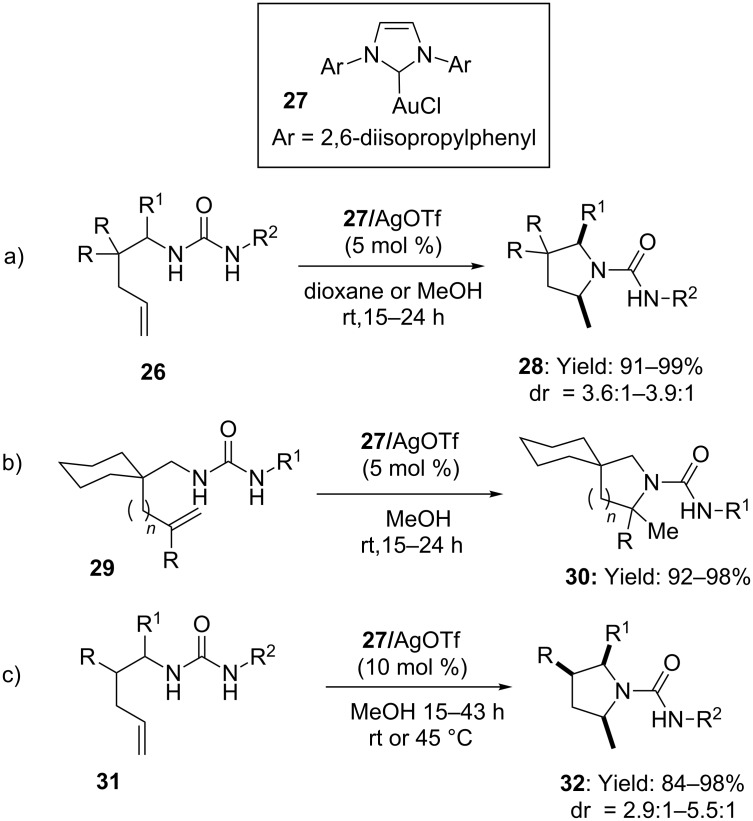
Intramolecular hydroamination of *N*-alkenylureas catalyzed by gold(I) carbene complex.

In addition, the introduction of carbene ligands [44] allowed performing the intramolecular hydroamination of *N-*alkenyl ureas, efficiently (Scheme 9) [45]. When IPrAuCl (**27**) and AgOTf were mixed together in 1:1 ratio, the intramolecular *exo-*addition of *N’-*alkyl and *N’-*arylurea to primary or secondary olefins took place smoothly at room temperature to afford the variously substituted pyrrolidines **28** and piperidines **30** (Scheme 9a,b). Even unsubstituted substrate **31** afforded the corresponding product **32** in excellent yield although more forcing conditions were necessary (Scheme 9c). Monosubstitution at carbon C1 or C2 of the chain led to the formation of the product 2,4-*cis* or 2,5-*cis-***32** with diastereomeric ratios up to 5.5:1.

**Scheme 10:**
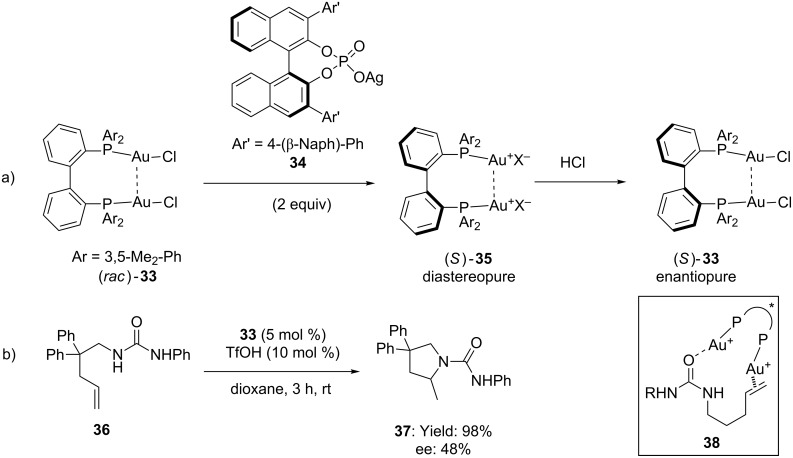
Enantioselective hydroamination of alkenyl ureas with biphenyl tropos ligand and chiral silver phosphate.

Enantioselective variant of this transformation was recently reported by Mikami and co-workers [46]. The protocol focuses on the use of a gold complex comprising the racemic 2,2'-bis(diphenylphosphino)-1,1'-biphenyl digold(I) complex **33** which, in combination with enantiopure silver phosphate **34**, afforded the diastereopure complex **35**. Interestingly, the strong [Au(I)]–[Au(I)] aurophilic interaction in **35** prevented racemization even if chiral anions were removed (Scheme 10a). (*S*)*-***33** found application in the intramolecular hydroamination of **36**, delivering **37** in quantitative yield (98%) and moderate enantioselectivity (ee up to 48%, Scheme 10b). The efficiency of **33** was ascribed to double activation of the substrate by the binuclear gold complex **38**.

**Scheme 11:**
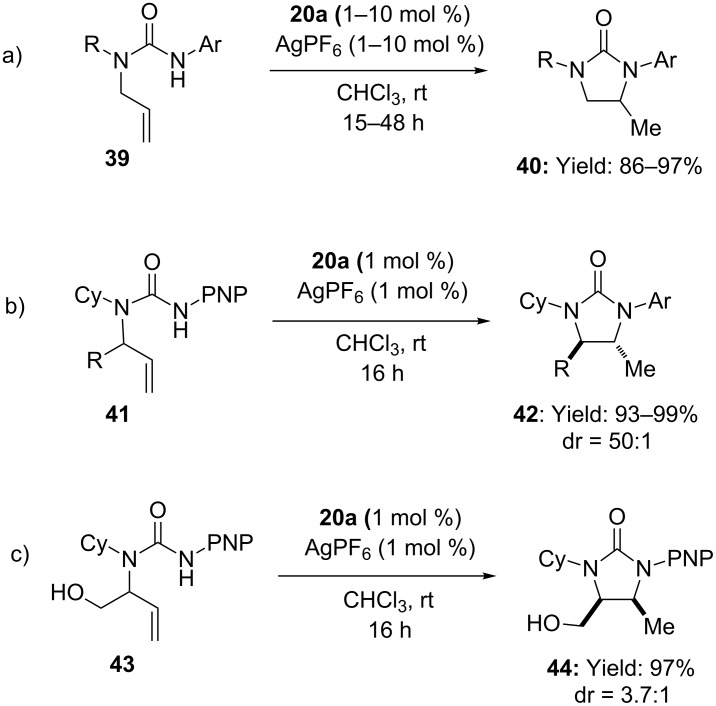
Intramolecular [Au(I)]-catalyzed hydroamination of N-allyl-N’-aryl ureas. (PNP = pNO_2_-C_6_H_4_, PMP = pMeO-C_6_H_4_).

The synthetic versatility of alkenyl ureas was further demonstrated by Widenhoefer employing slightly different substrates, namely *N-*allyl-*N’-*arylureas. When **39** was treated with a 1:1 mixture of **20a** and AgPF_6_, imidazolidin-2-ones **40** were obtained in high yields under mild reaction conditions (Scheme 11a) [47]. Analogously, substituted *N-*allylureas **41** provided **42** with high *trans* diastereoselectivity (dr *=* 50:1, Scheme 11b). Surprisingly, inversion of diastereoselection (i.e. *cis* stereoisomer as the major product) was recorded in the case of hydroxymethyl derivative **43**.

**Scheme 12:**
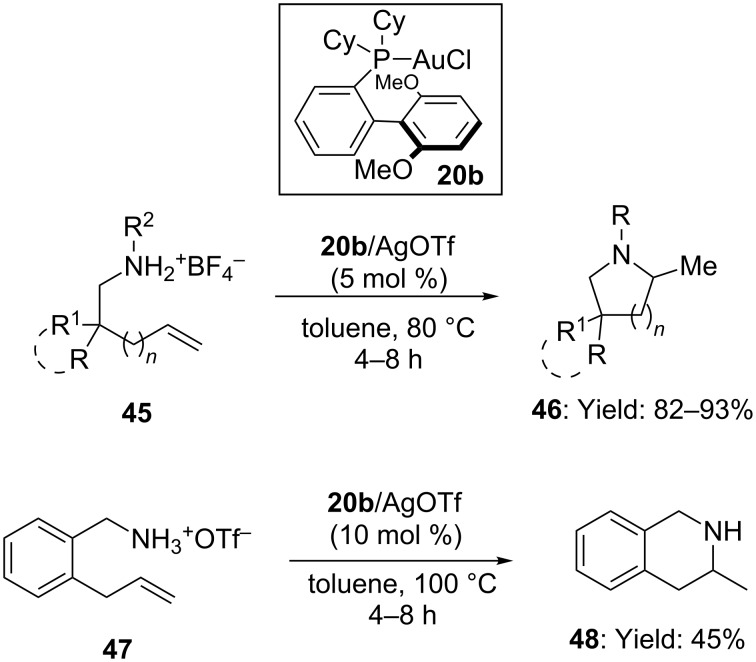
[Au(I)]-catalyzed hydroamination of alkenes with ammonium salts.

The challenging task of direct hydroamination with simple amines was faced, by means of in situ protection of the basic functionality as an ammonium salt (Scheme 12) [48]. The best catalyst for the titled reaction turned out to be the complex **20b** bearing an electron-rich phosphinic ligand. When **20b** was mixed with AgOTf (1:1 ratio) the intramolecular hydroamination of alkenyl ammonium salts **45**/**47** took place at 80–100 °C affording pyrrolidines and piperidines **46**/**48** in moderate to good yields.

**Scheme 13:**
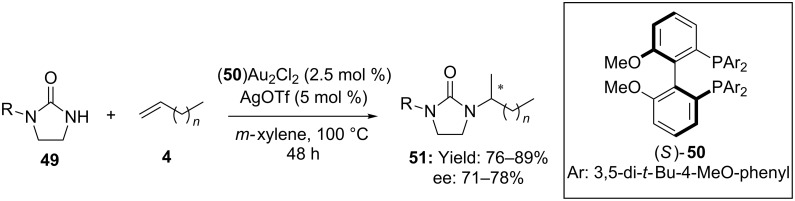
Enantioselective [Au(I)]-catalyzed intermolecular hydroamination of alkenes with cyclic ureas.

In 2009 Widenhoefer reported the first example of enantioselective gold-catalyzed intermolecular hydroamination of simple olefins. Firstly, the authors demonstrated that functionalization of ethylene derivatives could be conveniently accessed by using cyclic ureas **49** as nucleophiles in combination with phosphine gold complexes. Starting from this consideration a protocol for the enantioselective addition of cyclic ureas to simple alkenes **4** was developed (Scheme 13) [49]. In spite of the forcing conditions (100 °C for 48 hours) the use of the chiral binuclear gold complex (*S*)-**50**(AuCl)_2_ ensured enantioselectivity up to 78% for the products **51**.

A considerable enhancement of enantioselectivity was recently obtained by Toste’s group in the [Au(I)]-catalyzed intramolecular addition of sulfonamides to dienes (Table 2) [50]. The authors observed that, when the binuclear gold complex based on (*R*)-DTBM-segphos ligand **53** and AgBF_4_ was used in combination with alcoholic additives, the rate of the intramolecular hydroamination of **52** increased considerably, leading to the concomitant formation of **55** in combination with the expected product **54**. A screening of various chiral alcohols and kinetic studies established that the use of 2.0 equivalents of (−)-menthol led to an increase in both the yield and ee of **55** leaving unaffected the formation of **54**.

**Table 2:** Cooperative [Au(I)]/menthol catalysis for the enantioselective intramolecular hydroamination of dienes.

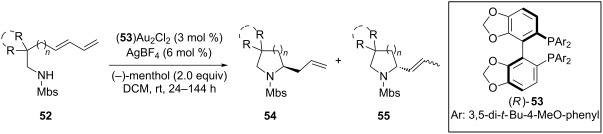				

Entry	**52**	R^a^	Yield (%)^b^ (54:55)	ee (%)^c^

1	a	Me	95 (1:6.3)	95
2	b	H	91 (1:6.1)	85
3	c	Ph	93 (1:3.5)	84
4	d	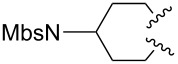	99 (1:5.0)	89
5	e	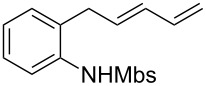	80 (1:8.0)	97
6	f	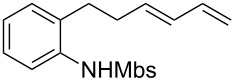	77 (1:12.1)	97
7	g	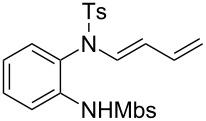	42 (1:3.2)	98
8	h	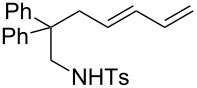	99 (1:1.5)	94
9	i	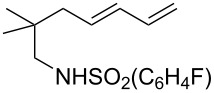	67 (1:7.6)	91

^a^Mbs = *p-*MeO-benzenesulfonyl. ^b^Combined yield of **54** and **55. **^c^The ee values are referred to (*E*)-**55**.

**Scheme 14:**
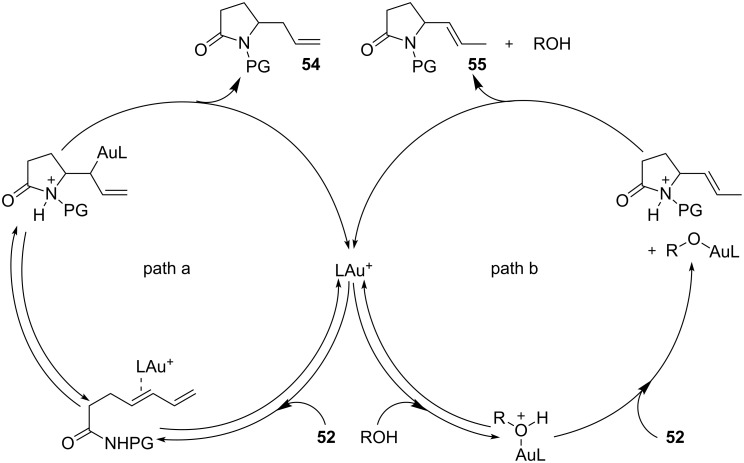
Mechanistic proposal for the cooperative [Au(I)]/menthol catalysis for the enantioselective intramolecular hydroamination of dienes.

This observation found adequate rationale with the presence of two different competing mechanisms for the formation of the products. In particular, while **54** was formed via a standard hydroamination reaction (Scheme 14, path a), compound **55** was formed via Brønsted acid catalysis because of the enhancement of alcohol acidity due to gold coordination (Scheme 14, path b). Under these conditions various pyrrolidines and piperidines were obtained in excellent yields and high enantioselectivity (Table 2).

### 4 Formation of C–C bonds

Besides heteroatoms, the electrophilic activation of alkenes by gold catalysts can be exploited also to form new C–C bonds. Early achievements relied on the use of active methylene compounds or electron rich arenes as carbon nucleophiles.

#### 4.1 Alkylation of active methylene compounds

**Scheme 15:**
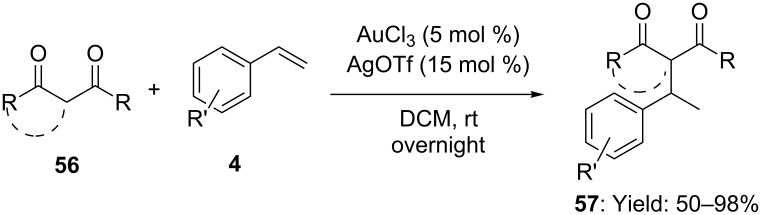
[Au(III)]-catalyzed addition of 1,3-diketones to alkenes.

Hydroalkylation of styrenes, indene and norbornene with 1,3-diketones was firstly reported in 2004 by using AuCl_3_ and AgOTf in 1:3 ratio as the catalyst (Scheme 15) [51]. The addition took place with Markovnikov selectivity in good yields, however optimal conditions suffered from severe limitations on 1,3-diketones as nucleophiles and unfunctionalized olefins. The same catalytic system was then employed with cyclic enols, dienes and trienes although lower yields were recorded [52].

**Scheme 16:**
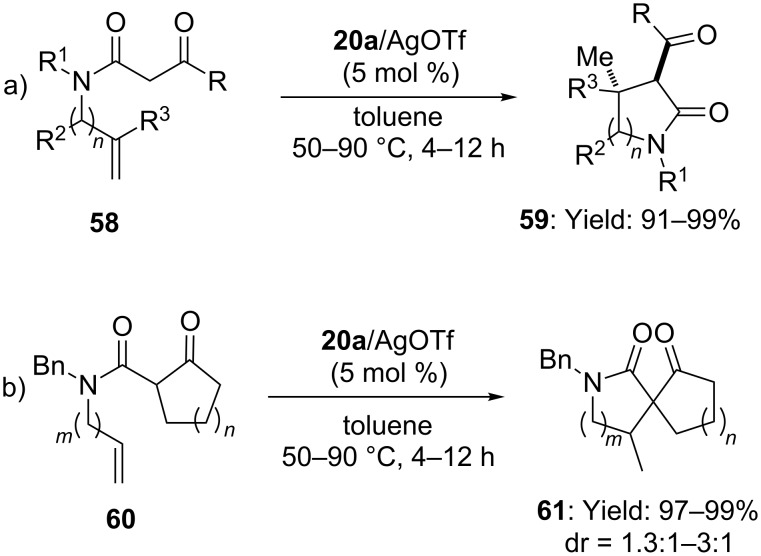
[Au(I)]-catalyzed intramolecular addition of β-keto amides to alkenes.

Remarkably, the intramolecular [Au(I)]-catalyzed addition of β-keto amides to inactivated alkenes was also exploited for the synthesis of highly substituted lactams **59**, also in a preparative scale [53]. When alkenyl β-keto amides **60** were reacted with a 1:1 mixture of phosphine-gold complex **20a** and AgOTf (toluene, 50–90 °C), *exo-trig* hydroalkylation of the C–C double bond took place, affording 5-, 6-membered lactams and spiro-lactams **61** in excellent yields and high *trans* diastereoselectivity (Scheme 16).

#### 4.2 The hydroarylation reaction

**Scheme 17:**
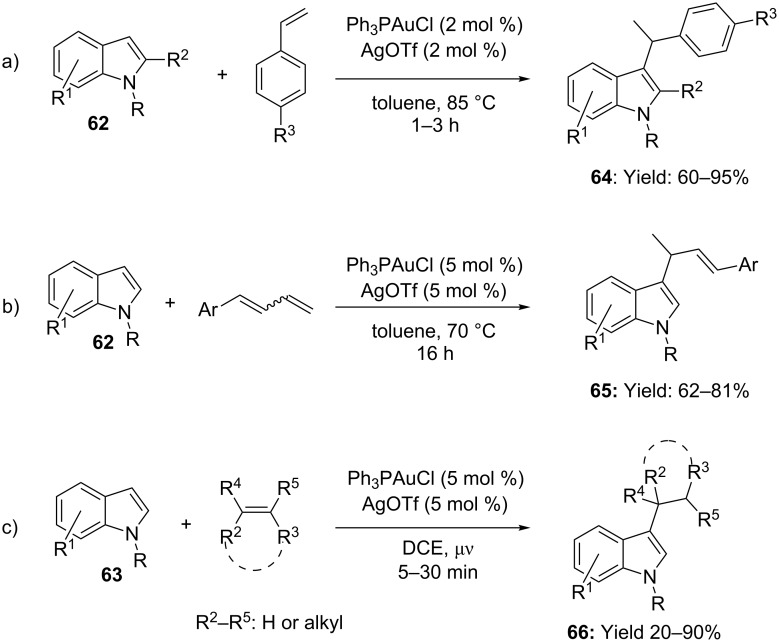
Intermolecular [Au(I)]-catalyzed addition of indoles to alkenes.

In addition to methylene active compounds, electron-rich benzenes and heteroaromatics can be added to gold activated alkenes under suitable conditions. In this context, in situ made [Ph_3_PAuOTf] was found to be an efficient catalyst for the addition of indoles **62** to styrenes and aryldienes in toluene at 85 °C (Scheme 17a,b). Less reactive aliphatic alkenes required harsher reaction conditions, however the corresponding hydroarylation products **66** were obtained from moderate to good yields under microwave irradiation (DCE, Scheme 17c) [54].

**Scheme 18:**
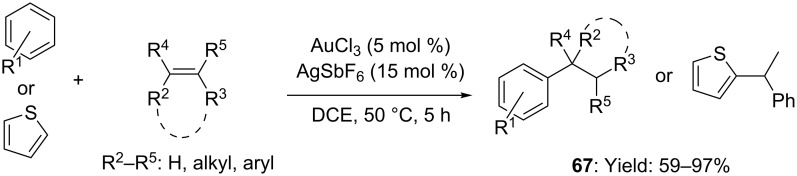
Intermolecular [Au(III)]-catalyzed hydroarylation of alkenes with benzene derivatives and thiophene.

Additionally, the stronger Lewis acid AuCl_3_/AgSbF_6_ was documented to catalyze the Markovnikov selective hydroarylation of aryl- and alkyl olefins with less-nucleophilic benzene derivatives and thiophene in good yields (Scheme 18, DCE, 50 °C) [55]. To be underlined that despite of efficiency, isomerization of the C=C was found competing with the desired Friedel–Crafts-type alkylation under the optimized reaction conditions.

**Scheme 19:**
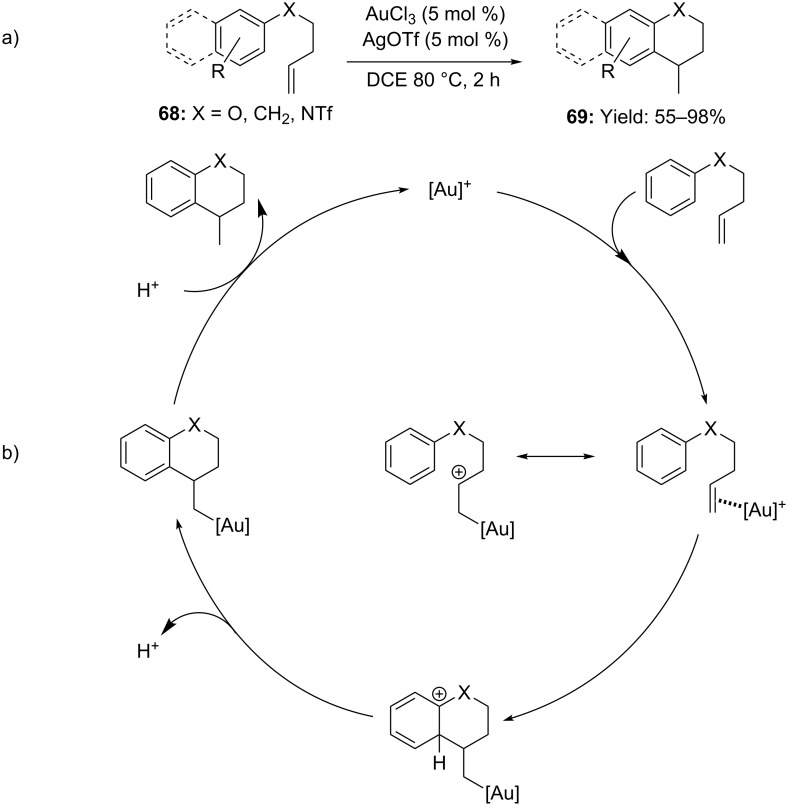
a) Intramolecular [Au(III)]-catalyzed hydroarylation of alkenes. b) A S_E_Ar-type mechanism was hypothesized by the authors.

Analogously, [Au(III)] catalysis was employed in the intramolecular hydroarylation of olefins. It is woth mentioning, that a 1:1 mixture of AuCl_3_ and AgOTf promoted the ring-closing processes of arenes **68**, delivering the corresponding dihydrobenzopyrans, tetralins and tetrahydroquinolines **69** in good yields (Scheme 19a). Experimental controls with deuterium labelled compounds suggested the step-wise mechanism described in Scheme 19b. In detail, initial gold activation of the olefin would trigger the outer-sphere attack of the aryl ring to the double bond followed by protodeauration of the alkyl–gold compound [56].

#### 4.3 Latest developments

**Scheme 20:**
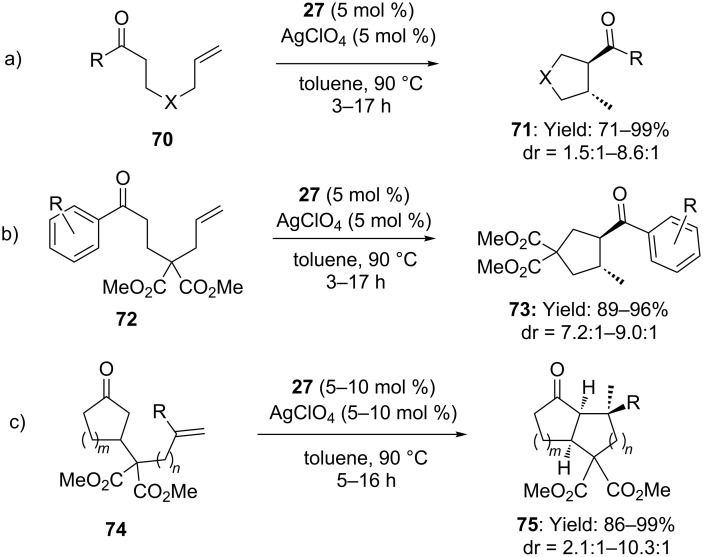
Intramolecular [Au(I)]-catalyzed hydroalkylation of alkenes with simple ketones.

Recent advances in the field of C–C bond forming processes through gold activated olefins allowed expanding the scope of the reaction to other carbon nucleophiles. For examples Che and co-workers reported on the hydroalkylation of alkenes with simple ketones [57]. Reaction of alkenyl ketones with IPrAuCl/AgClO_4_ (5 mol %) afforded functionalized cyclopentyl and cyclohexyl derivatives in excellent yields and good *trans* diastereoselectivity, via *exo-trig* cyclization (Scheme 20a–c).

**Scheme 21:**
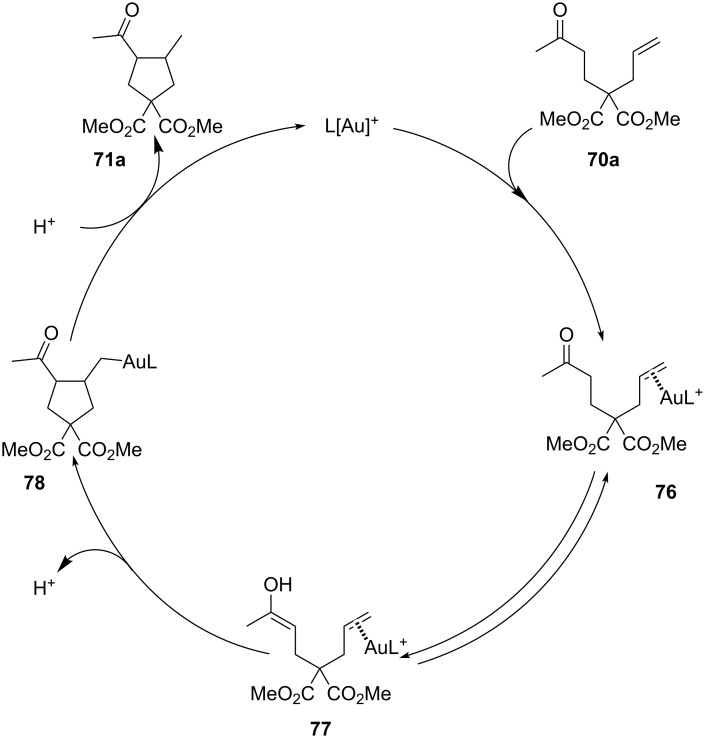
Proposed reaction mechanism for the intramolecular [Au(I)]-catalyzed hydroalkylation of alkenes with ketones.

The proposed mechanism involved activation of the C–C double bond by the carbene-based cationic gold species to form the intermediate **76** which underwent gold-promoted tautomerization affording the enol **77**. Nucleophilic attack of the enol on the gold activated alkenes led to a new C–C bond with subsequent releasing of the product **71** (Scheme 21).

**Scheme 22:**
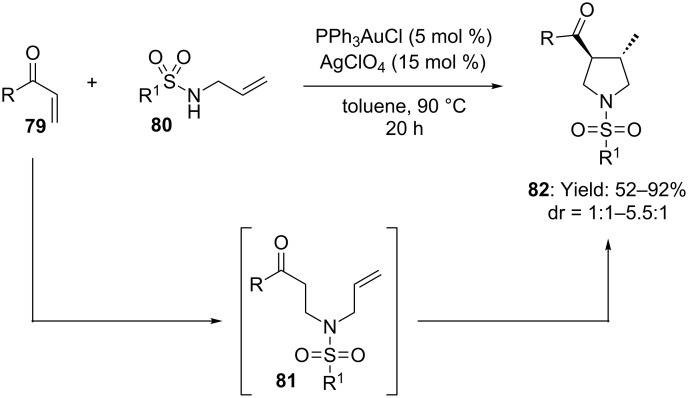
Tandem Michael addition/hydroalkylation catalyzed by [Au(I)] and [Ag(I)] salts.

This protocol was also extended to an intermolecular variant (i.e. one-pot *N*-Michael addition/hydroalkylation). The use of [PPh_3_AuCl/AgClO_4_] (5/15 mol %) furnished corresponding functionalized pyrrolidines in good yields and moderate stereoselectivity (Scheme 22) [58].

**Scheme 23:**
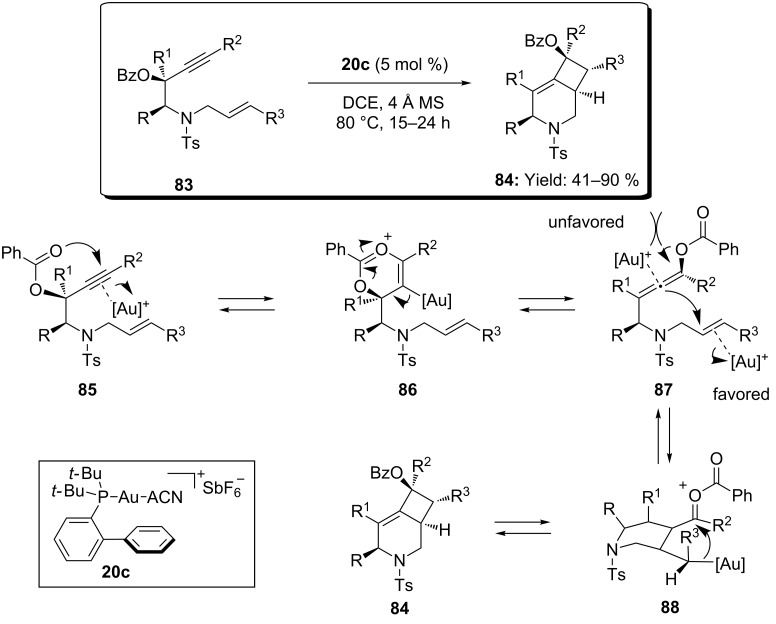
Intramolecular [Au(I)]-catalyzed tandem migration/[2 + 2] cycloaddition of 1,7-enyne benzoates.

The preferred alkene activation versus allenes was recently observed in the cascade 1,3-migration/[2 + 2] cycloaddition of 1,7-enyne benzoates (Scheme 23) [59]. When **83** was heated in presence of the silver free gold complex **20c** a variety of highly substituted azabicyclo[4.2.0]oct-5-enes **84** was obtained in good yields as a single regio- and diastereoisomer. The protocol displayed excellent functional group compatibility and efficient transfer of chirality was observed with enantiopure substrates. The proposed mechanism proceeded through 3,3-migration of the propargylic ester to form the allenoate **87**. The expected activation of the allene was probably unfavoured for steric reasons, therefore gold activation of the alkene moiety triggered attack of the more nucleophilic double bound of the allene, forming the intermediate **88**. Finally, condensation of the alkylgold **88** onto the carbonyl group led to the bicyclic product **84** (Scheme 23).

**Scheme 24:**
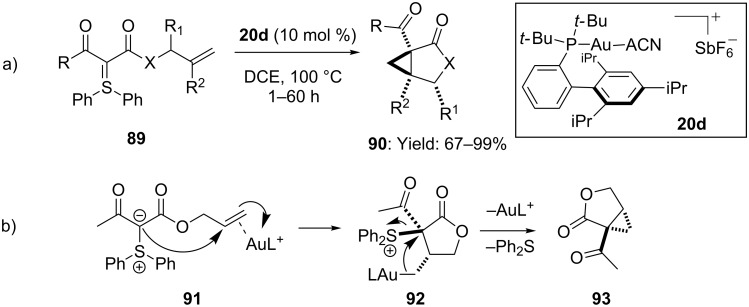
Intramolecular [Au(I)]-catalyzed cyclopropanation of alkenes.

An interesting example of gold-catalyzed intramolecular cyclopropanation of olefins was recently documented by Maulide and coworkers [60]. In particular, a range of densely functionalized heterobicyclic and carbocycles **90** were readily accessible in high yields and high stereoselectivity starting from properly functionalized sulfonium ylides **89** (Scheme 24). Computational and experimental investigations suggested the initial **20d**-based electrophilc activation of the C=C, with consequent nucleophilic attack by the ylidic carbon onto the internal carbon of the double bond. Finally, the intermediate lactone **92** underwent cyclopropanation, delivering SPh_2_ as a leaving group (Scheme 24b).

### 5 Addition to allylic alcohols

The use of simple allylic alcohols as alkylating agents, in place of more activated analogues (i.e. halides, acetates, carbonates and phosphates) is highly desirable from a synthetic, environmental and economic point of view [61]. Late transition metal catalysts demonstrated efficiency in addressing the poor reactivity of these substrates [62]. Due to the ability of gold complexes to act as σ- and π-acids, gold catalysis gained a prominent role in the activation of allylic alcohols, delivering of water as the side-product. As the topic has been recently extensively reviewed by Aponick and Biannic [7], only the most recent examples of gold-catalyzed manipulation of inactivated allylic alcohols will be discussed here [63–64].

#### 5.1 Mechanistic considerations

**Scheme 25:**
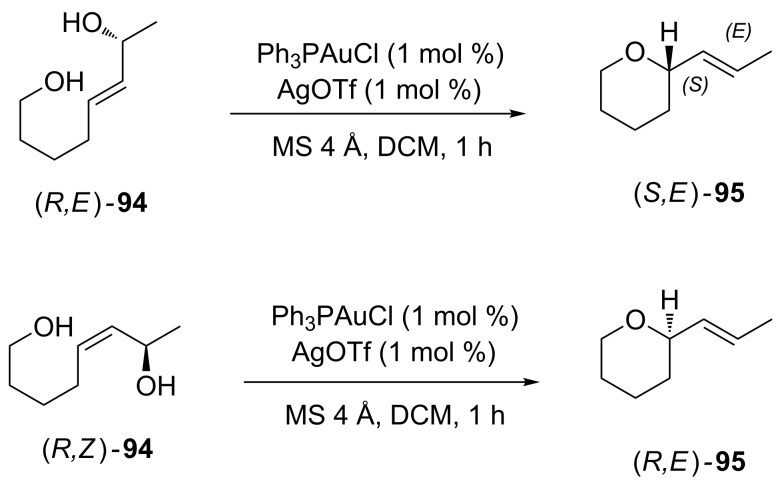
Stereospecificity in [Au(I)]-catalyzed hydroalkoxylation of allylic alcohols.

Very recently Ess and Aponick reported a detailed mechanistic study on the intramolecular hydroalkoxylation of hydroxy allylic alcohols **94**, pointing out the key role of intramolecular hydrogen bond (H-bond) interactions for both reactivity and stereoselectivity [65]. Theoretical calculations and experimental evidences ruled out the S_N_1 reaction mechanism via allylic carbocation. Experiments with stereodefined hydroxy allylic alcohols revealed that the reaction was stereospecific: alcohols with the same configuration of the secondary carbinol atom, but different geometry of the double bond, afforded the cyclized product **95** with the opposite absolute configuration of the stereocenter, but the olefin had *E* configuration in both cases (Scheme 25).

**Scheme 26:**
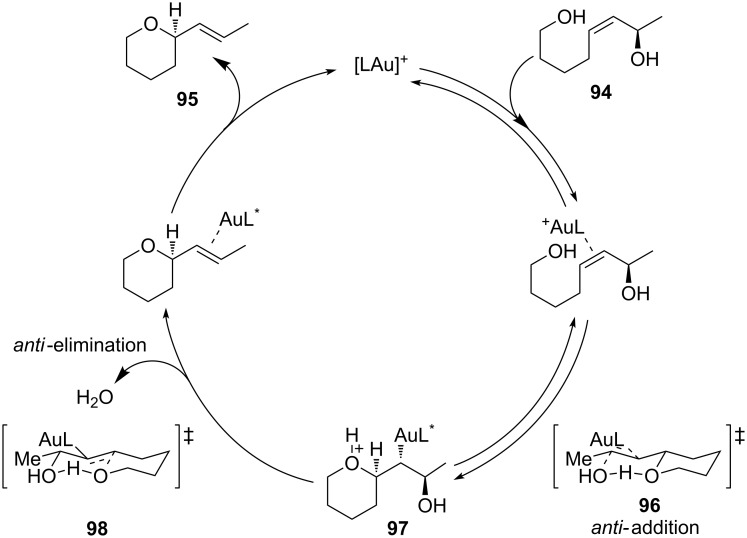
Mechanistic investigation on the intramolecular [Au(I)]-catalyzed hydroalkoxylation of allylic alcohols.

Whereas concerted S_N_2’ pathway was judged energetically unfavourable, two possible mechanistic channels were supposed to account for the observed stereoselectivity: *anti* addition followed by *anti*-elimination or *syn* addition followed by *syn-*elimination. Calculations showed that the former pathway was energetically favoured. Interestingly, a strong hydrogen bond was established during the catalytic cycle between the hydroxy groups. The H-bond turned out to be a key interaction for the high reactivity of **94** toward cyclization, leading to an intramolecular proton transfer resulting into a better leaving group. Furthermore, as the H-bond was conserved during the entire course of the reaction, it also determined the *E* geometry of the newly formed C–C double bond (Scheme 26).

**Scheme 27:**
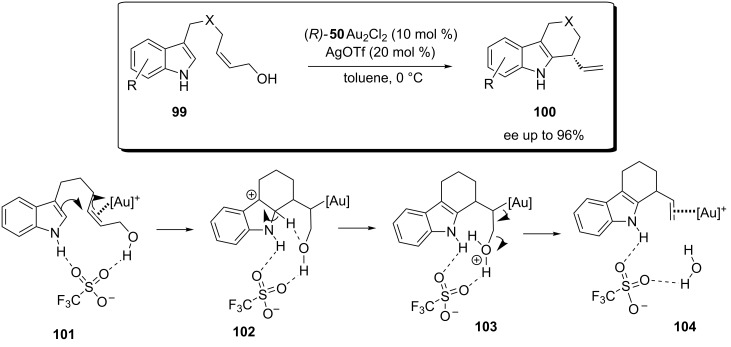
Mechanistic investigation on the intramolecular enantioselective [Au(I)]-catalyzed alkylation of indoles with allylic alcohols.

The importance of the H-bond network in gold-catalyzed enantioselective transformations of allylic alcohols has been recently highlighted also by Bandini and Miscione [66–68] that documented on the intramolecular enantioselective allylic alkylation of indoles. Combined computational and experimental studies revealed the presence of a complex H-bond network between the indole ring, the counterion (i.e. OTf^−^) and the leaving OH group along all the reaction profile (Scheme 27). Multiple functions were recognized in these interactions. Firstly, all the strong H-bonds conferred a constrained conformation to the substrate (U-fold*)* placing the two reacting moieties in close proximity (**101**). Secondly, after nucleophilic attack of the indole ring, the counterion facilitated the elimination of water (**103**) by shuttling one proton atom, from the indolyl ring to the leaving hydroxy group. The proposed catalytic cycle accounted also for the observed stereochemistry of the product **100** (*R*) when the chiral binuclear gold complex (*R*)*-*DTBM-MeO-biphep **50** was employed.

#### 5.2 Recent advances

Latest advances in gold-catalyzed transformations of allylic alcohols mainly concern the development of stereoselective processes. In particular, mild conditions allowed a more efficient development of enantioselective protocols and the scope of the process can be widen to more functionalized substrates, efficiently.

**Scheme 28:**
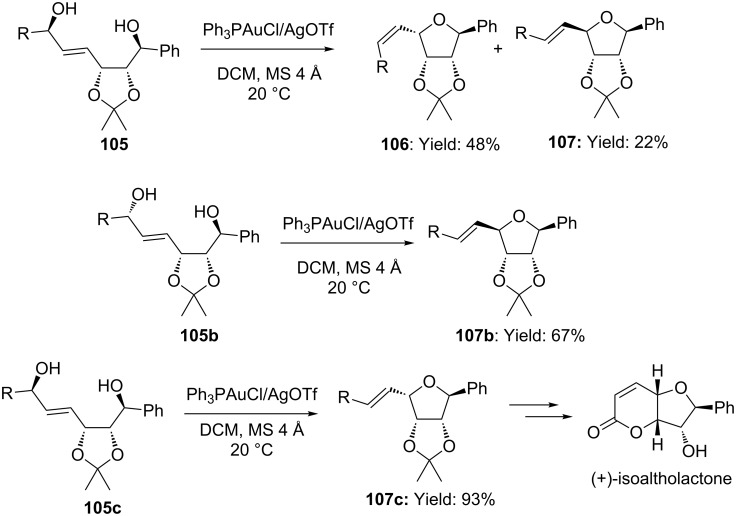
Synthesis of (+)-isoaltholactone via stereospecific intramolecular [Au(I)]-catalyzed alkoxylation of allylic alcohols.

For examples Robertson and co-workers reported on the synthesis of (+)-isoaltholactone, by adopting an intramolecular stereoselective Ph_3_PAuOTf*-*catalyzed *5-exo* alkoxylation of allylic alcohols, to form tetrahydrofuran rings (Scheme 28) [69]. Experiments with configurationally defined hydroxy allylic alcohols **105b**,**c** demonstrated the stereospecificity of the process. Due to the complexity of the substrates, the stereochemical model proposed by Aponick [65] could only partially account for the stereochemical outcome of the reaction, since the importance of the configuration of the carbon bearing the nucleophilic OH group was not considered.

**Scheme 29:**
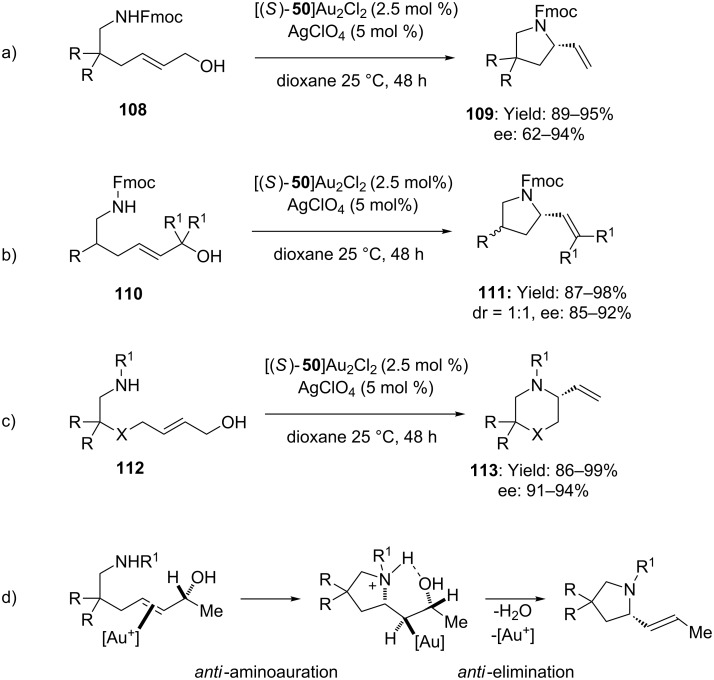
Intramolecular enantioselective dehydrative amination of allylic alcohols catalyzed by chiral [Au(I)]-complexes.

In this segment, Widenhoefer has recently documented on the enantioselective dehydrative amination of allylic alcohols with carbamates [70–71]. Pyrrolidines and piperidines **109** were obtained with enantiomeric excesses up to 94% by using a chiral gold complex based on (*S*)-DTBM-MeO-biphep (Scheme 29). Mechanistic investigation highlighted complete catalyst control on the configuration of the newly formed stereocenter. On the contrary, studies on stereochemically defined secondary allylic alcohols revealed that the configuration of the allylic carbon atom affected the configuration of the newly formed C–C double bond. The *E* configuration of the starting material turned out to be fundamental to achieve high enantioselectivity. Also in this case, a mechanism sketch consisting on *anti*-aminoauration followed by *anti-*elimination was invoked to account for the observed experimental outcome (Scheme 29d).

**Scheme 30:**
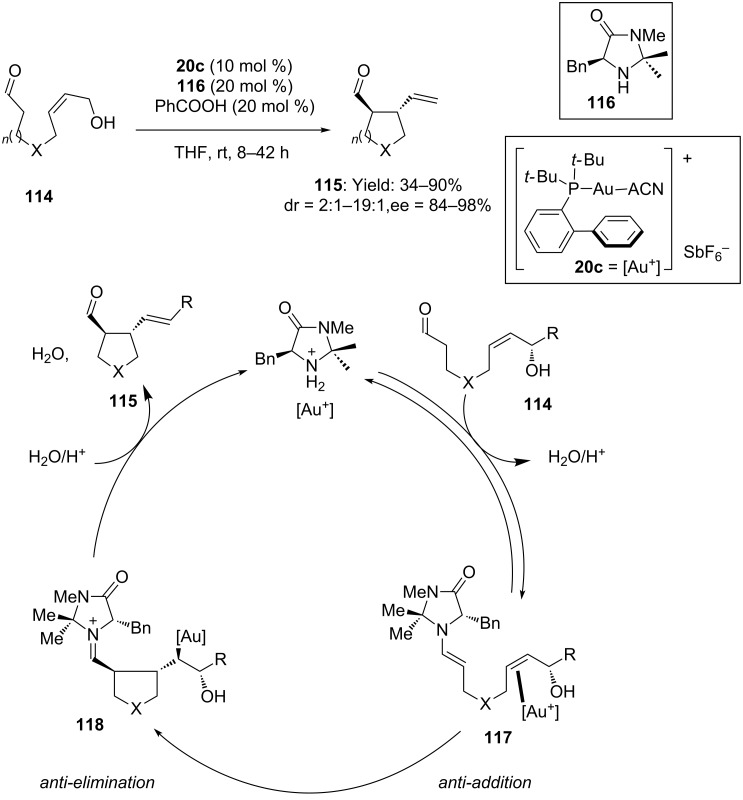
Enantioselective intramolecular hydroalkylation of allylic alcohols with aldehydes catalyzed by **20c** and chiral secondary amine.

A complementary approach for the enantioselective synthesis of 3,4-disubstituted pyrrolidines was recently reported by Bandini and co-workers [72]. Intramolecular hydroalkylation of allylic alcohols with aldehydes was performed under synergistic activation of the substrate by gold catalyst **20c** and organocatalyst **116** (Scheme 30). The 5-membered hetero- and carbocycles **115** were obtained in moderate to good yield and interesting level of diastereo- and enantioselectivity, supporting the perfect compatibility between the amino-catalyst and the electrophilic gold complex. Experiments on chiral enriched secondary alcohols suggested the *anti*-attack of the in situ formed enamine **117** on gold activated allylic alcohol followed by *anti-*elimination of water.

### 6 Double functionalization of olefins by oxidative strategies

Differently from many transition metals, usually gold is not subjected to redox processes during the catalytic cycles. However, in recent years it has been demonstrated that both [Au(I)/Au(III)] (i.e. monometallic catalytic systems) and [Au(I)Au(I)/Au(II)Au(II)] (i.e. bimetallic catalytic systems) redox couples could be accessed using external stoichiometric oxidants (i.e. Selectfluor or hypervalent iodine compounds) [73]. As schematically shown in Figure 1 this approach allows a double functionalization of simple alkenes with subsequent formation of new C–X and C–C bonds in a single catalytic process.

**Scheme 31:**
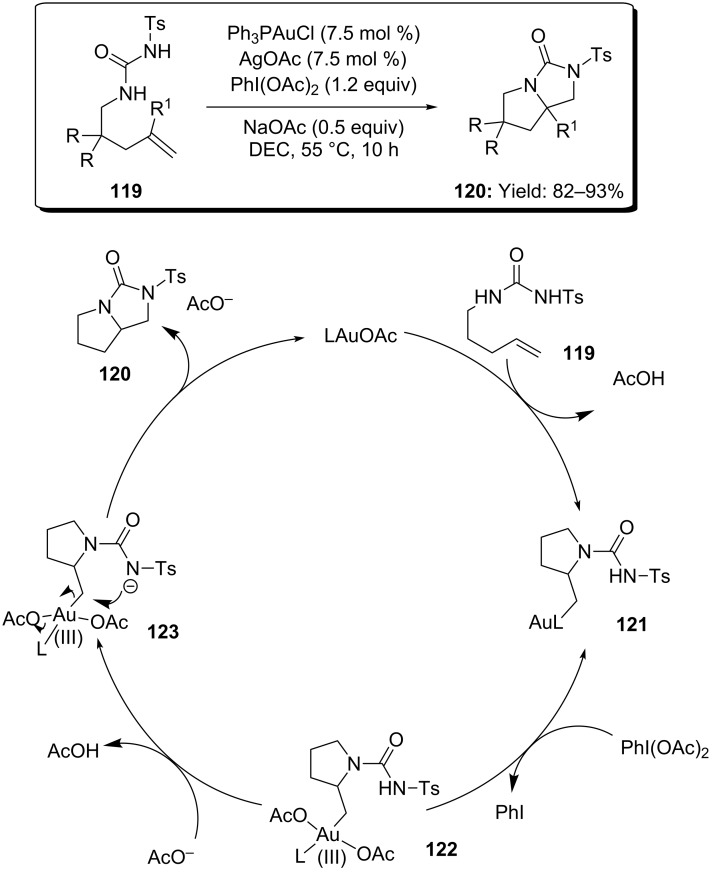
Gold-catalyzed intramolecular diamination of alkenes.

In 2009 Muñiz and coworkers reported on the diamination of alkenes catalyzed by Ph_3_PAuOAc in presence of PhI(OAc)_2_ (Scheme 31) [74]. The process led to the formation of two new C–N bonds under mild conditions with excellent yields. A mechanistic rationale was also provided dealing with initial [Au(I)]-promoted *anti-*amination with formation of alkylgold intermediate **121**, followed by oxidation to [Au(III)] species **122** by means of PhI(OAc)_2_. A final ring-closing step will provide product **120** (Scheme 31).

**Scheme 32:**
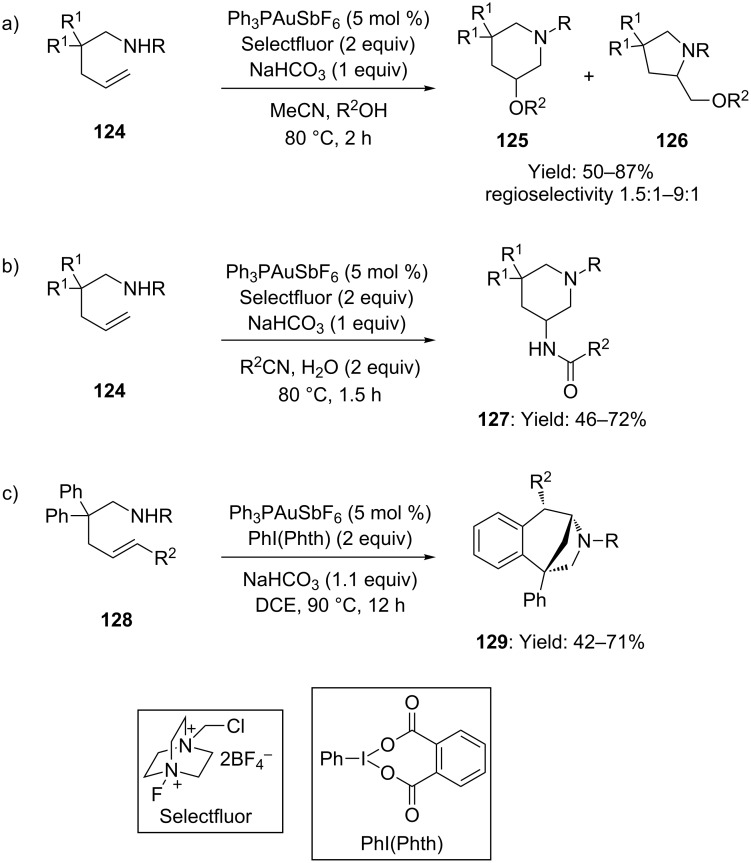
Gold-catalyzed aminooxygenation and aminoarylation of alkenes.

Exploiting a similar strategy, Nevado developed a flexible protocol for the gold-assisted aminooxygenation of alkenes, intercepting the hydroamination intermediate with various nucleophiles (i.e. alcohols, ethers and esters, Scheme 32a) [75]. A remarkable variety of products could be accessed with minimal variation of the reaction conditions. For instance, by using nitriles as solvents and only 2 equivalents of water, a nucleophilic attack of the nitrile itself on the hydroamination intermediate took place, affording the corresponding aminoamidation product **127** in moderate to good yields (Scheme 32b). In absence of external nucleophiles, the phenyl rings of the substrate backbone could intercept the hydroamination intermediate affording tricyclic 3-benzazepines **129** (Scheme 32c).

**Scheme 33:**
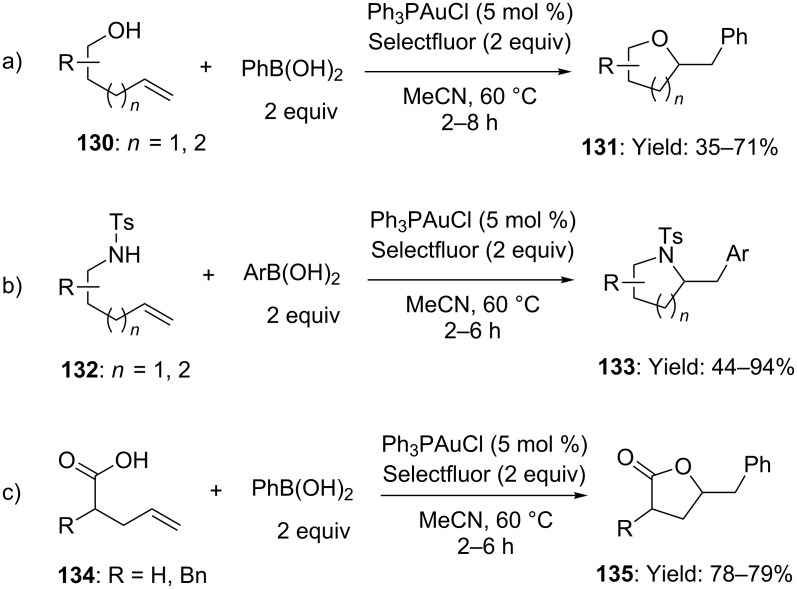
Gold-catalyzed carboamination, carboalkoxylation and carbolactonization of terminal alkenes with arylboronic acids.

In the same field, Zhang reported the carboamination, carboalkoxylation and carbolactonization of terminal alkenes with arylboronic acids. Under best conditions, oxidative gold catalysis provided expedient access to various substituted *N*- or *O*-heterocycles in high yields (Scheme 33) [76]. Deuterium labeling experiments unambiguously demonstrated the *anti-*functionalization of the double bond and the use of neutral gold complexes suggested that [Au(I)] oxidation took place prior of C–X bond formation.

**Scheme 34:**
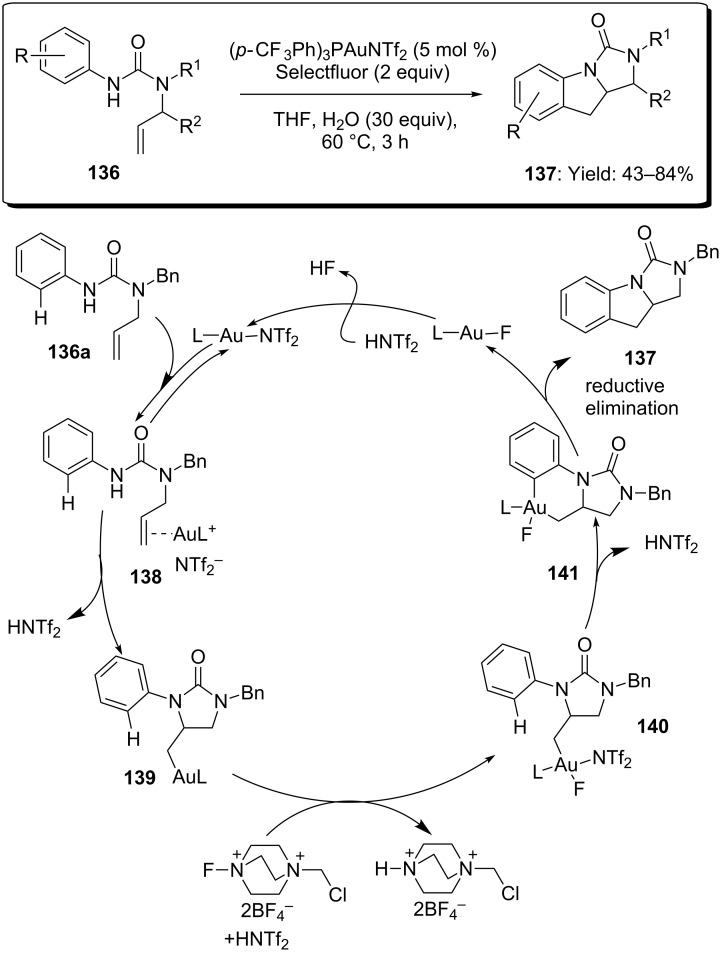
Synthesis of tricyclic indolines via gold-catalyzed formal [3 + 2] cycloaddition.

The same team also reported on the efficiency of gold catalysis in the functionalization of C–H bonds [77]. In particular, *N-*aryl-*N’-*allylureas **136** were selectively transformed into tricyclic indolines **137** in good yields. The reaction preceeded through the regioselective *5-exo anti-*aminoauration of the C–C double bond (**139**) followed by oxidative coupling of the formed alkylgold moiety with the *ortho* C–H bond of the tethered phenyl group. Compound **137** was finally obtained via reductive elimination of intermediate **141** (Scheme 34). The formal [3 + 2] annulation between the aniline moiety and the C–C double bond constitutes the first example of C–H functionalization by alkylgold intermediates. The optimized conditions required the use of the electrophilic complex [(*p-*CF_3_Ph)_3_PAuNTf_2_] and 30 equivalents of water, in order to increase the solubility of Selectfluor in THF.

**Scheme 35:**
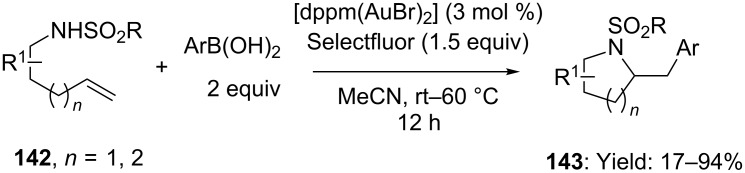
Gold(I) catalyzed aminoarylation of terminal alkenes in presence of Selectfluor [dppm = bis(diphenylphosphino)methane].

Heteroarylation of alkenes with arylboronic acids under the assistance of redox gold catalysis was also elegantly investigated by Toste’s group. In particular, aminoarylation of terminal olefins was documented in the presence of catalytic amounts of [dppm(AuBr)_2_] (3 mol %) and Selectfluor as the stoichiometric oxidant [78]. Despite the undoubted synthetic interest relying on the synthetic approach (a wide range of densely functionalized nitrogen-based heterocyclic cores (**143**) were readily accessible) (Scheme 35), intriguing mechanistic insights derived from a detailed investigation based on experimental and computational observations [79].

**Scheme 36:**
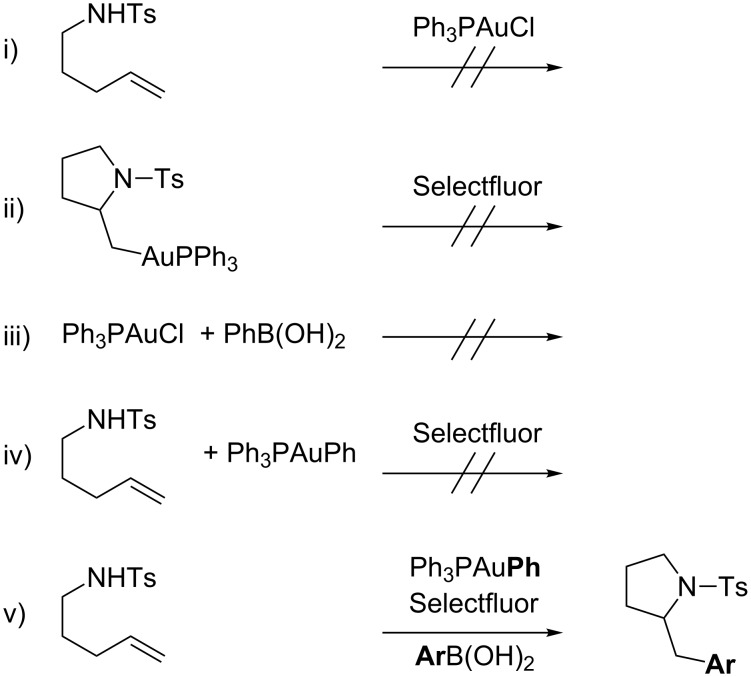
Mechanistic investigation on the aminoarylation of terminal alkenes by bimetallic gold(I) catalysis in presence of Selectfluor.

In particular, the inability of [Au(I)] halide complexes in promoting the aminoauration of the double bond suggested the oxidation of the [Au(I)] complex as the first step of the catalytic cycle (Scheme 36 i). This hypothesis was further supported by the failure recorded when an isolated alkylgold compound was reacted with Selectfluor and PhB(OH)_2_ (ii). Moreover, Ph_3_PAuCl and PhB(OH)_2_ were mixed together, no formation of Ph_3_PAuPh was detected ruling out the transmetallation as initial stage of the catalytic cycle (iii). In addition, Ph_3_PAuPh proved to be a competent catalyst of the process but only in the presence of arylboronic acid (iv and v). The latter evidence prompted the authors to conclude that the aryl group was transferred from the boronic acid and not from the gold complex (i.e. the phenyl group on the in situ formed PPh_3_AuPh complex acts as a spectator in the process).

**Scheme 37:**
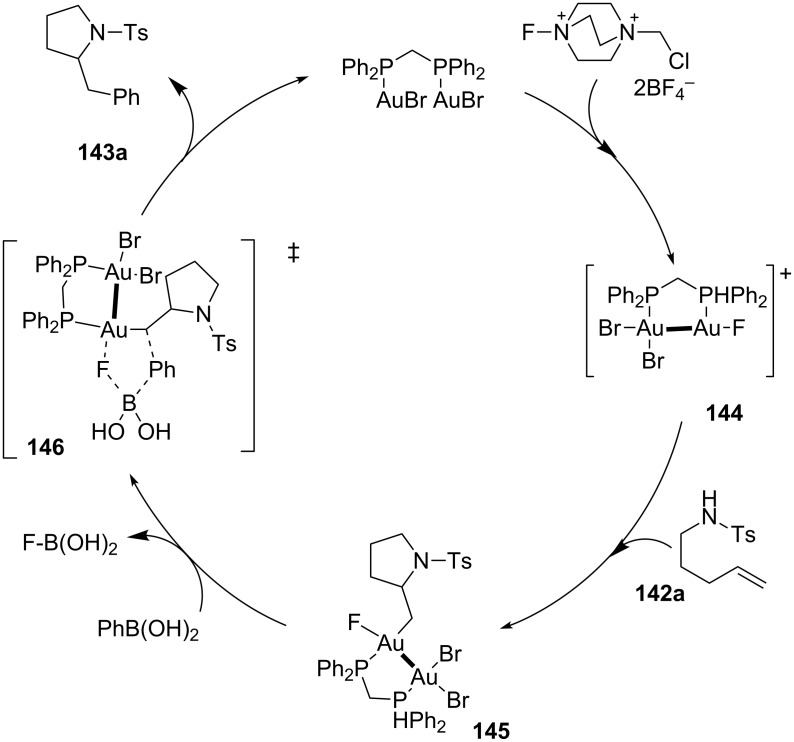
Proposed mechanism for the aminoarylation of alkenes via [Au(I)-Au(I)]/[Au(II)-Au(II)] redox catalysis.

Based on such observations, the mechanistic cycle depicted in the Scheme 37 was proposed. Oxidation of the bimetallic [dppm(Au_2_Br_2_)] complex by Selectfluor led to the preferred formation of the cationic [Au(II)Br_2_–Au(II)F]^+^ complex **144** also due to the instauration of a strong aurofilic interaction (σ-bond) between the two gold atoms. Then, intramolecular aminoauration of the C=C occurred via an *anti-*stereochemical reaction profile, leading to intermediate **145**. Finally, arylation of **145** was supposed to occur via a concerted bimolecular elimination (**146**), in which the F–B bond assisted the formation of the new C–C bond (Scheme 37) [80].

It should be emphasized that the latter step of the proposed reaction machinery referred as “redox synergy” is in clear conflict with the commonly reported alternative invoking transmetalation/reductive elimination steps (see Schemes 33 and 34). The catalytic superiority of bimetallic systems with respect to PPh_3_AuOTf in the aminoarylation is clearly explainable by the latter mechanistic proposal, that found solid validations/analogies in the “Pd---Pd” cooperative catalysis [81].

**Scheme 38:**
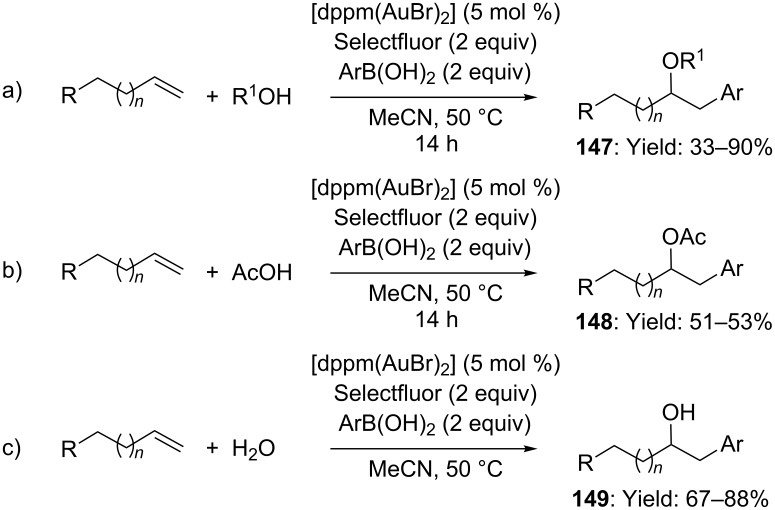
Oxyarylation of terminal olefins via redox gold catalysis.

The same catalytic system was also utilized in the three-component oxyarylation of olefins (Scheme 38) [82]. The reaction took place under mild conditions and exhibited a wide substrate scope, being highly tolerant towards a number of olefins, arylboronic acids and nucleophiles. In particular, primary, secondary, tertiary alcohols and even water could be employed as nucleophiles, affording the corresponding ethers and alcohols in moderate to good yields (up to 90%, Scheme 38a,c). Differently, although carboxylic acids were suitable nucleophiles as well, the corresponding products were isolated in lower extent (Scheme 38b).

**Scheme 39:**
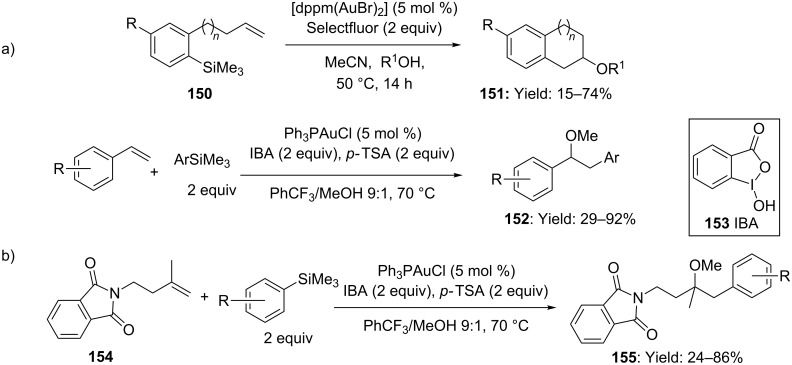
a) Intramolecular gold-catalyzed oxidative coupling reactions with aryltrimethylsilanes. b) Oxyarylation of alkenes catalyzed by gold in presence of iodine-(III) compound IBA as an external oxidant.

Furthermore, the present strategy was extended to arylsilanes, enabling the use of oxygen- and nitrogen-containing coupling partners [83–84]. Interestingly, due to the employment of Selectfluor as a stoichiometric exogenous oxidant, the addition of basic activators for silane reagents were not required. The ready availability of silane precursors, with respect to boronic acid counterparts, allowed an intermolecular variant to be successfully developed (Scheme 39a).

A slight modification of the reaction conditions enabled to expand the oxidative coupling to a wider range of olefins. In particular the use of IBA (**153**) as the oxidant allowed substrates incompatible with Selectfluor, (i.e. styrene and *gem-*disubstituted olefins) to be efficiently employed (Scheme 39b) [85].

**Scheme 40:**
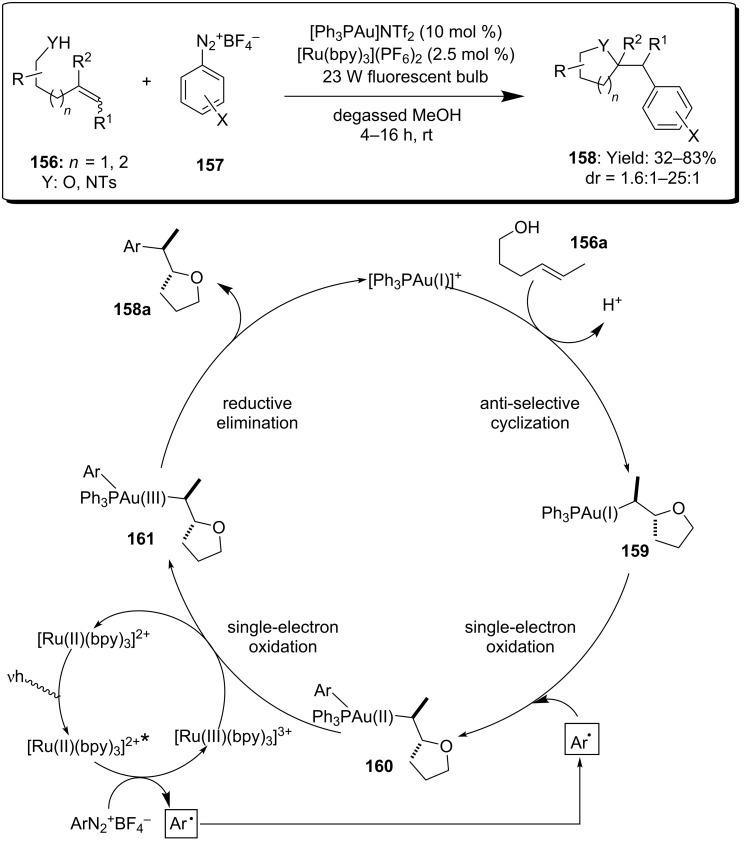
Oxy- and amino-arylation of alkenes by [Au(I)]/[Au(III)] photoredox catalysis.

An innovative approach to the double functionalization of olefins was developed by Glorius and co-workers, very recently. The authors reported on the use of visible light-mediated photoredox catalysis to access the [Au(I)]/[Au(III)] redox couple during the intramolecular oxy- and aminoarylation of alkenes (Scheme 40) [86].

Optimal conditions for the reaction involved the use of Ph_3_PAuNTf_2_ in presence of [Ru(bpy)_3_]^2+^ as redox photocatalyst and aryl diazonium salts **157**. In the proposed tandem catalytic cycle, the initial *anti* oxy-auration of the double bond led to the alkylgold intermediate **159**, that was oxidized to [Au(II)] **160** via a SET process by the aryl radical formed in the photoredox catalytic cycle. The highly reactive species **160** was promptly oxidized by [Ru(III)(bpy)_3_]^3+^ affording the [Au(III)] intermediate **161** and regenerating the [Ru(II)(bpy)_3_]^2+^ photocatalyst. Finally, arylated tetrahydrofuran **158** was obtained by reductive elimination with concomitant regeneration of the [Au(I)] catalyst.

## Conclusion

Metal catalyzed electrophilic activation of isolated alkenes is by far considered among the most challenging metal-assisted nucleophilic manipulation of inactivated unsaturated hydrocarbons. Relative inertness of C=C with respect to alkynes or allenes accounts for this trend. In this scenario [Au(I)] and [Au(III)] catalysis is playing a major role leading to tremendous developments spanning from C-/hetero-nucleophilic manipulations, mono-/difunctionalizations, intra-/intermolecular transformations and regio-/stereoselective methodologies. In this chemistry, it should be mentioned that concomitant catalytic activity exerted by Brønsted acidity can not be ruled out a priori and it should not be underestimated. Therefore, practitioners should carefully determine the real role of gold complexes during the reaction course, with respect to [H^+^] sources, from time to time. Experimental controls would certainly contribute to unambiguously clarify the intrinsic mechanistic aspects of the process but they would also concur to a better optimization/rationalization of optical outcomes deriving from stereoselective transformations. Despite the apparent interchangeability between cationic gold(I) species and [H^+^] sources, a major breakthrough introduced by [Au(I)] complexes in the nucleophilic manipulation of inactivated alkenes relies on enantioselective processes, that still represent an unsolved issues in metal-free catalysis [87].
